# Comparative molecular docking and simulation analysis of molnupiravir and remdesivir with SARS-CoV-2 RNA dependent RNA polymerase (RdRp)

**DOI:** 10.6026/97320630017932

**Published:** 2021-11-30

**Authors:** Shashank M Patil, KR Maruthi, Shrisha Naik Bajpe, VM Vyshali, S Sushmitha, Chagalamari Akhila, Ramith Ramu

**Affiliations:** 1 Department of Biotechnology and Bioinformatics, School of Life Sciences, JSS Academy of Higher Education and Research, Mysuru-570015, Karnataka, India; 2 Department of Biotechnology, Sri Dharmasthala Manjunatheshwara College (Autonomous), Ujire-574240, Karnataka, India; 3 Department of Biotechnology, BMS College for Women, Basavangudi, Bengaluru-560004, Karnataka, India; 4 Department of Pharmacology, JSS Medical College, JSS Academy of Higher Education and Research, Mysuru-570015, Karnataka, India

**Keywords:** SARS-CoV-2, RdRp, Molnupiravir, Remdesivir, in silico experiments, molecular docking, molecular dynamics, binding free energy calculations, druglikeliness, pharmacokinetic properties

## Abstract

Treatment of SARS-CoV-2 targeting its RNA dependent RNA polymerase (RdRp) is of current interest. Remdesivir has been approved for the treatment of COVID-19 around the world. However, the drug has been linked with pharmacological limitations like adverse effects and reduced efficiency. Nevertheless, recent advancements have depicted molnupiravir as an effective therapeutic agent to target the SARS-CoV-2 RdRp. The drug has cleared both in vitro and in vivo screening. It is in phase-III clinical trial. Nonetheless, there are no data on themolecular binding interaction of molnupiravir with RdRp. Therefore, it is of interest to report the binding interaction of molnupiravir using molecular docking. It is also of interest to show its stability during interaction using molecular dynamics and binding free energy calculations along with drug likeliness and pharmacokinetic properties in comparison with remdesivir.

## Background:

SARS-CoV-2 is currently regarded as one of the most detrimental diseases with no available therapeutic options. Even though few vaccines are employed to prevent the pandemic, no specific therapeutic agents have been discovered till date [1,^2^]. Several hundreds of phytochemicals and chemically synthesised compounds have been reported to induce anti-viral activity against SARS-CoV-2 through targeting different viral enzymes and proteins [3,4]. Among these targets, RNA dependent RNA polymerase serves as one of the chief targets, being responsible for the viral genome replication. It is coded by a non-structural protein known as nsp-14. It is linked withcofactors like as nsp7 and nsp8 to perform polymerase activity. It's made up of a nsp12 core catalytic unit coupled to a nsp7-nsp8 (nsp8-I) heterodimer, as well as an extra nsp-8 (nsp8-II) subunit [5].

Several medicines including remdesivir, ritonavir, lopinavir, favipiravir, ribavirin, sofosbuvir, and ivermectin, have been used to limit viral genome replication. In addition to chemotherapy, cellular therapeutics such as CAR T cells has been employed to stop the viral genome from replicating [3]. The backbone of current antiviral medication is nucleoside analogues working through this mechanism, with over thirty licenced (either alone or in combination) for the prophylaxis and treatment of viral infections. Including remdesivir, these ribonucleoside analogues when integrated into nascent chain RNA, they selectively serve as competitive alternative substrates, interrupting viral genomic and/or mRNA production [6]. Remdesivir has been widely used over the world and has been licenced for global use due to its higher efficacy than the other medications. However, remdesivir has been found with few adverse effects like cognitive delirium, kidney injury, nausea, vomiting, and rectal bleeding [5].

In course of these developments, it becomes essential to either to repurpose the current chemotherapeutics or to design novel drugs. However, Recent studies have highlighted molnupiravir (MK-4482/EIDD-2801) as a strong ribonucleoside analogue that creates interference in viral genome replication.The drug is reported to be in phase 2 clinical trial [6,7].According to biochemical tests, RdRp utilizes molnupiravir as a substrate instead of cytidine triphosphate or uridine triphosphate. Molnupiravir directs inclusion of either G or A in the RNA. When the RdRp uses the resultant RNA as a template, it results in the synthesis of modified RNA products. Molnupiravir may form stable base pairs with either G or A in the RdRp active centre, explaining how the polymerase escapes proofreading and synthesises altered RNA, producing mutagenesis products [8-10].Molnupiravir inhibits the virus with a potency 27,5 times that of remdesivir in human cell culture, for which it has to be given intravenously. The lung viral load in animals lowers 25,000 times after oral administration and roughly 100,000 times when given as prophylactic. It stops the virus from spreading and eliminates its presence in the oropharynx [11,12]. In case of human trials, two variants of the virus including Wuhan strain or B.1.1.7 and B.1.351 have been tested with molnupiravir. However, the drug was found to be effective against infections caused by each of the variations, suggesting that it could be used to combat present and future virus variants [13-15]. Inspired by these outcomes, an outline of large-scale synthesis of the drug has also been suggested [16]. Therefore, it is of interest to report the binding interaction of molnupiravir using molecular docking. It is also of interest to show its stability during interaction using molecular dynamics and binding free energy calculationsalong with druglikeliness and pharmacokinetic properties in comparison with remdesivir.

## Materials & Methods:

### Molecular docking simulation:

This computational prospection involves the pipeline of experiments previously designed by the authors [5]. SARS-CoV-2 RdRp's crystal structure was retrieved from the RCSB PDB database (PDB ID: 6M71) [17]. Whereas, 3D structures of molnupiravir and remdesivir were obtained from PubChem database [18]. AutoDock Tools 1.5.6 was used to prepare the protein and ligand structures for docking simulation [19]. Protein and ligand preparation for the docking process were performed according to the previous study conducted by the authors [20]. Briefly, protein structure was cleaned to remove water and heteroatoms, followed by stabilizing with the addition of hydrogen atoms. It was further energy-minimized by adding Kollmann and Gasteiger charges. Ligand preparation was done by adding Kollmann and Gasteiger charges and keeping the ligands with default torsions. For molecular docking simulation, an open source command line software known as AutoDock Vina 1.2 designed for the docking of the molecular entities was used [21]. It uses the Broyden-Fletcher-Goldfarb-Shanno (BGFS) algorithm for the perturbation and local optimization of ligands into the target site, and evaluates the scoring function of each ligand conformation. Out of 10 binding positions generated for each ligand, the first position with the zero root mean square deviation (RMSD) of atomic positions was considered to be highly valid, as it possesses the most negative binding affinity, indicating stronger binding interaction. The visualization of docking simulation was done using BIOVIA Discovery Studios Visualizer 2021, an open source visualizing GUI software [20].

### Molecular dynamics simulation:

From the docking results, the first conformations of bound molnupiravir and remdesivir were obtained and subjected to molecular dynamics simulation according to the previous study conducted by the authors [20]. For this, a command-line interface biomolecular software package, GROMACS 2021.3 was used [22]. Both the protein-ligand complexes were approximated with CHARMM27 forcefield, and ligand topology was obtained using SwissParam server [23]. After the initial addition of hydrogen atoms, the systems were vacuum-minimized using the steepest descent algorithm. This was followed by the incorporation of solvent and subsequent neutralization using counter ions to maintain the 0.15 M salt concentration. Both the protein-molnupiravir(10,563 residues) and protein-remdesivir(10,583 residues) were energy-minimized using the steepest descent and conjugate gradient methods. This was followed by the NVT and NPT ensembles (1000 ps with the dt of 2 fs) and MD run (100 ns with the dt of 2 fs) using the leapfrog integrator algorithm at 310 K temperature and 1 bar pressure. The coordinates and energies of the system were saved at every 10 ps. Molecular dynamics simulation trajectories included protein-ligand complex root-mean square deviation (RMSD), root-mean square-fluctuation (RMSF), ligand RMSD, and ligand hydrogen bonds. Visualization of these molecular dynamics simulation results was done with XMGRACE, plot-analysing GUI software [20].

### Binding free energy calculations:

Based on the outcomes of molecular dynamics simulation, binding free energy calculations were performed both molnupiravir-protein and remdesivir-protein complexes. Molecular Mechanics/Poisson-Boltzmann Surface Area (MM-PBSA) approach was used to calculate binding free energy for each ligand-protein complex using the g_mmpbsa tool, which uses GROMACS trajectories as input [24]. The binding free energy is estimated in the g_mmpbsa programme using three components: molecular mechanical energy, polar and apolar solvation energies. The binding free energy was calculated using the molecular dynamics trajectories of the last 50 ns (of both dihydroquercetin and acarbose) and dt 1000 frames. The binding free energy is computed using the equation (I), whereas the free energy of each complex component is calculated using the equation (II). Further, equation (III) is used to evaluate average potential energy in vacuum, where Ebonded represents bond length, angle and torsion angle, and Enonbonded represents van der Waals and electrostatic energies. Equation (IV) is given to calculate the energy needed to transfer the solute from vacuum to the solvent. The Gpolar and Gnonpolar indicate electrostatic and non-electrostatic support to the solvation free energy, respectively as described earlier [5,20].

### Druglikeliness and pharmacokinetic studies:

For the druglikeliness and pharmacokinetic studies, the chemical structures of both molnupiravir and remdesivirwere submitted to the ADMETlab server (https://admetmesh.scbdd.com/) in SMILES format for this investigation.For the druglikeliness evaluation, Lipinski's rule of five was considered. In case of pharmacokinetic studies, parameters like Madin-Darby Canine Kidney (MDCK)permeability,P-glycoprotein (P-gp) inhibitor, protein-plasma binding (PPB), volume distribution (VD), cytochrome P (CYP) inhibition, clearance (CL), human Ether-a-go-go-Related Gene (hERG), and AMES carcinogenicity test were considered.

## Results and Discussion:

Molecular docking simulation was performed to understand the interaction of molnupiravir and remdesivir at molecular level. Molnupiravir was predicted with comparatively more negative (-7.3 kcal/mol) (stronger) binding affinity than remdesivir (-6.9 kcal/mol). It bound to the finger subdomain of the RdRp using 6 hydrogen bonds including ASP 452 (2.87 Å), ARG 553 (2.56 Å), ARG 553 (2.52 Å), LYS 621 (2.08 Å), CYS 622 (2.41 Å), and PRO 620 (3.33 Å). In addition, it also formed 2 electrostatic bonds with ARG 624 (4.93 Å), ASP 623 (3.54 Å), and one hydrophobic pi-alkyl bond with ARG 624 (5.49 Å). Molnupiravir also bound to the palm subdomain of the enzyme through a hydrogen bond with ASP 760 (2.36 Å). However, remdesivir was predicted to bind with finger subdomain using 3 hydrogen bonds with TYR 619 (2.04 Å), ASP 623 (2.68 Å), and ASP 760 (3.61 Å). It also formed 4 electrostatic bonds with ASP 623 (4.10 Å), ARG 553 (4.04 Å), ASP 618 (3.89 Å), ASP 618 (4.53 Å) and 2 hydrophobic pi-alkyl bonds with LYS 621 (5.05 Å) and ARG 624 (5.33 Å). Remdesivir also bound with palm subdomain with a pi-alkyl bond with LYS 798 (5.21 Å). Although both ligands occupied the same binding site, interaction of molnupiravir found to be better than remdesivir due to the more number of hydrogen bonds. However, remdesivir was also predicted with 2 unfavourable hydrogen bonds with ARG 553 (4.35 Å) and ASP 623 (2.77 Å) Figure 1(see PDF) and 2 indicate the visualization of docking simulation of molnupiravir and remdesivir, respectively.

Molecular dynamics simulation is performed to know the stability of the ligands inside the binding pocket of the enzyme through the analysis of a variety of trajectories that precisely predict the stability of the ligands bound to the protein during the simulation. They include protein-ligand complex RMSD, RMSF, ligand RMSD, and ligand hydrogen bonds. In this study, The RMSD plot of the protein-ligand complex displays the stability of the ligand inside the binding pocket over the course of a 100 ns simulation. The RMSD trajectories of protein-backbone atoms and protein-molnupiravir complex were found to get stable after 20 ns, whereas protein-remdesivir complex was found to be stable after 40 ns, free from initial fluctuations. However, the protein-remdesivir complex was predicted with irregular fluctuations throughout the simulation process. The average deviation of a particle (e.g., a protein residue) from a reference location over time is calculated using RMSF. As a result, RMSF focuses at the regions of the protein structure that deviate the most/least from the mean. In case of protein-backbone atoms, the fluctuations were observed at C-terminal region (after 850 residues) and loop regions (400 – 600 residues). However, compared to the protein-remdesivir complex, the protein-molnupiravir complex was predicted with lesser number of fluctuations significantly after 200 residues. To determine the stability of ligands inside the binding pocket, RMSD of both 6i and acarbose was plotted. In the ligand RMSD plot analysis, it was found that the RMSD of molnupiravir was consistent throughout the simulation without fluctuation and equilibrated at ∼0.75 nm. In case of remdesivir the plot was found to be stable during few intervals (10 - 30 ns, 40 - 80 ns, and 85 - 100 ns). Unlike molnupiravir, remdesivir showed comparatively more fluctuations during the simulation. Further, it is essential to calculate the number of H-bonds formed during the simulation, as few bonds were simultaneously broken and rebuilt. In case of both remdesivir and molnupiravir, the number of hydrogen bonds formed during molecular dynamics simulation were found to be the same (n = 5). By the virtue of the simulation process, it can be concluded that molnupiravir is comparatively more stable than remdesivir in terms of binding interaction with the SARS-CoV-2 RdRp. Figure 3(see PDF) denotes the multiple trajectories of the simulation process that run for 100 ns.

Binding free energy calculations are calculated to understand the extent of binding interaction of ligands with the target proteins during molecular dynamics simulation through different energy parameters including Van der Waal's, electrostatic, polar solvation, SASA, and binding energies. In this study, protein-molnupiravir complex was predicted to be more stable than protein-remdesivir complex. Both complexes were formed majorly using Van der Waal's energy. It was also found that SASA energy was the least contributor for the formation of both the complexes. In addition, standard deviations of all types of energies show that remdesivir deviate from the original values. These results predict that remdesivir is comparatively unstable than molnupiravir during the formation of protein-ligand complex. A summary of the binding free energy calculations has been given in Table 1(see PDF).

To evaluate the oral bioavailability of potential drug candidates in silico, druglikeliness and pharmacokinetic analyses, also known as ADMET (adsorption, distribution, metabolism, excretion, and toxicity) predictions, are used. Molnupiravir was predicted with no violation of Lipinski's rule. However, remdesivir had 3 violations including higher molecular weight (602.58 g/mol), number of hydrogen bond acceptors, and number of rotatable bonds. In case of ADMET properties, both compounds showed positive results for MDCK permeability, P-gp inhibition, protein-plasma binding volume distribution, and CYP inhibition. However, remdesivir found with negative results for clearance (CL), hERG, and AMES toxicity tests. These results predict that remdesivir might be associated with toxic and carcinogenic properties, while molnupiravir was found to show no toxicity and carcinogenicity. Details of the druglikeliness and pharmacokinetics evaluation are given in Table 2(see PDF). Figure 4(see PDF) describes the pharmacokinetic map of both molnupiravir and remdesivir.

Note: Molecular weight (<500: good); Topological polar surface area (<150: good); Number of rotatable bonds; Number of hydrogen bond acceptors (≤10: good); Number of hydrogen bond donors (≤5: good); MDCK cell permeability (low: < 2 x 10-6 cm/s, medium: 2-20 x 10-6 cm/s, high: > 20 x 10-6 cm/s); P-gp inhibition (> 0.0 or positive value: good);Protein-plasma binding (Optimal: < 90%. Drugs with high protein-bound may have a low therapeutic index); Volume distribution (Optimal: 0.04-20L/kg); Cytochrome P inhibition (> 0.0 or positive value: good); Clearance (High: >15 mL/min/kg; moderate: 5-15 mL/min/kg; low: <5 mL/min/kg); hERG inhibition (0.0 or negative value: good); AMES toxicity (0.0 or negative value: good).

In the absence of specific therapeutic options, SARS-CoV-2 continues to dwell around the world with its unmatchable pathogenic modifications that are rooted in its genome. Even in the presence of efficient vaccines, the virus is affecting the people worldwide through its adaptive modifications in the host body [number] [25,^26^, 27,^28^]. The RNA dependent RNA polymerase (RdRp) enzyme plays a crucial role in the production of the viral genome. Several therapeutics have already been tried to inhibit the RdRp in different stages including clinical trial on humans. Among such compounds, molnupiravir has been reported with extensive anti-viral activity till date [29, [30,31]. However, there have been no studies which elucidate the interaction and stability of molnupiravir with SARS-CoV-2 RdRp at molecular level. This prompts us to come out with a computational approach to understand the same.

During the molecular docking process,it was observed that even though both ligands bound to the same binding pocket, molnupiravir had the most negative (hence more efficient) binding interaction in comparison with remdesivir. In case molecular dynamics simulation, molnupiravir was predicted with higher stability and efficient binding interaction in comparison with remdesivir. In the process that was run for 100 ns, molnupiravir showed no abnormal fluctuations in all the parameters evaluated, which predicts the stability inside the binding pocket of the enzyme. However, remdesivir was predicted with fluctuations in case of protein-ligand complex and ligand alone RMSDs, and protein-ligand RMSF. Further, the binding free energy calculations also revealed the same, yet deciphering the cause of protein-ligand complex formation (Van der Waal's energy) and deviation of remdesivir from real values. Furthermore, in course of druglikeliness remdesivir failed to obey the Lipinski’s rule of five, which could be the reason behind its reduced oral bioavailability?

In a recent study [32], most of the RdRp inhibiting drugs including sofosbuvir, ribavirin, galidesvir, remdesivir, favipiravir, and tenofovir were assessed for their binding affinity. In comparison with all these drugs, molnupiravir was predicted with most negative binding affinity (-7.4 kcal/mol). Along with these, some other drugs like lopinavir, dexamethasone, merimpodib, and ritonavir were evaluated for their binding affinity with SARS-CoV-2 RdRp in a study [33]. Yet molnupiravir showed extensive binding affinity and interaction compared to these drugs. Further, two sets of repurposed chemotherapeutic medicines found through computational approaches have been tried against the SARS-CoV-2 RdRp, but they have failed druglikeliness and pharmacokinetic assays [34,35]. Furthermore, another an silico study conducted on different RdRp inhibiting drugs except molnupiravir, did not reveal the stability of the other drugs through dynamics simulations. The study even failed to provide an account for druglikeliness, pharmacokinetics, and toxicity parameters [36].In addition to the studies mentioned above, we have performed binding free energy calculations and ADMET studies which predict the stability and pharmacological potential of the drug candidates, which predict molnupiravir as one of the best inhibitor of SARS-CoV-2 RdRp.

## Conclusion:

We document the comparative molecular docking and simulation analysis of molnupiravir and remdesivirwith SARS-CoV-2 RNA dependent RNA polymerase (RdRp) for further consideration in the treatment against CoVid-19.
